# Association of Cerebrospinal Fluid Neurofilament Heavy Protein Levels With Clinical Progression in Patients With Parkinson Disease

**DOI:** 10.1001/jamanetworkopen.2022.23821

**Published:** 2022-07-26

**Authors:** Linbo Wang, Wei Zhang, Fengtao Liu, Chengjie Mao, Chun-Feng Liu, Wei Cheng, Jianfeng Feng

**Affiliations:** 1 Institute of Science and Technology for Brain-Inspired Intelligence, Fudan University, Shanghai, China; 2 Key Laboratory of Computational Neuroscience and Brain-Inspired Intelligence, (Fudan University), Ministry of Education, Shanghai, China; 3 MOE Frontiers Center for Brain Science, Fudan University, Shanghai, China; 4 Zhangjiang Fudan International Innovation Center, Shanghai, China; 5 Department of Neurology, Huashan Hospital North, Fudan University, Shanghai, China; 6 Department of Neurology and Clinical Research Center of Neurological Disease, The Second Affiliated Hospital of Soochow University, Suzhou, China; 7 Department of Computer Science, University of Warwick, Coventry, United Kingdom

## Abstract

**Importance:**

Neurofilament light in biofluids has been associated with progression of Parkinson disease (PD), but the association between neurofilament heavy (NfH) and progression of PD has not been investigated.

**Objective:**

To evaluate the associations of cerebrospinal fluid (CSF) NfH (cNfH) levels and motor and cognitive progression in PD.

**Design, Setting, and Participants:**

This cohort study used data from the Parkinson Progression Marker Initiative ranging from June 2010 to November 2018. Participants were recruited from 24 participating sites worldwide (United States, Europe, and Australia). Data were analyzed from October 20 to December 18, 2021.

**Exposures:**

Concentrations of NfH in CSF.

**Main Outcomes and Measures:**

The primary outcomes were Movement Disorder Society–sponsored revisions of the Unified Parkinson Disease Rating Scale (MDS-UPDRS) Part III; scores range from 0 to 132, with higher scores indicating worse motor function, and Montreal Cognitive Assessment (MoCA); scores range from 0 to 30, with higher scores indicating better cognitive function. The associations of cNfH levels and longitudinal change in MDS-UPDRS-Part-III and MoCA were examined using linear mixed-effects models with PD duration as the time scale. Partial correlation analysis was conducted to examine the associations of cNfH levels and α-synuclein, amyloid-β 1-42 (Aβ_42)_, phosphorylated tau at threonine 181 position (P-tau), and total tau (T-tau) levels in CSF.

**Results:**

A total of 404 patients with PD (mean [SD] age, 61.7 [9.7] years; 263 were men [65.1%]; within 2 years of diagnosis; Hoehn and Yahr <3) were included. Higher baseline cNfH levels were associated with greater increases in MDS-UPDRS Part-III (β = 0.39; 95% CI, 0.12-0.66; *P* = .003) and faster decreases in MoCA (β = −0.18; CI, −0.24 to −0.11; *P* < .001). Levels of cNfH were correlated with CSF levels of α-synuclein (Spearman *r* = 0.25; 95% CI, 0.15-0.34; *P* < .001), Aβ_42_ (Spearman *r* = 0.18; 95% CI, 0.08-0.27; *P* < .001), P-tau (Spearman *r* = 0.25; 95% CI, 0.15-0.35; *P* < .001), and T-tau (Spearman *r* = 0.31; 95% CI, 0.21-0.40; *P* < .001) at baseline.

**Conclusions and Relevance:**

Higher baseline cNfH levels were associated with faster motor and cognitive progression. This finding suggests that cNfH may be of some value for stratifying patients with PD who have different progression rates.

## Key Points

**Question:**

Are cerebrospinal fluid (CSF) neurofilament heavy (cNfH) levels associated with clinical progression in patients with Parkinson disease?

**Findings:**

In this cohort study of 404 patients with Parkinson disease, higher baseline cNfH levels were associated with faster worsening of motor and cognitive symptoms. In addition, cNfH levels were associated with levels of other CSF biomarkers (ie, α-synuclein, amyloid-β 1-42, phosphorylated tau at threonine 181 position, and total tau) at baseline.

**Meaning:**

These findings suggest that cNfH levels may be useful in stratifying patients with Parkinson disease who have different progression rates; however, this finding should be investigated further.

## Teaser

This cohort study assesses the association of cerebrospinal fluid neurofilament heavy protein levels with motor and cognitive disease progression among patients with Parkinson disease.

## Introduction

Axonal degeneration is an early pathologic process in Parkinson disease (PD).^1,2^ Neurofilaments, which are major cytoskeletal components of myelinated axons, are released into the extracellular fluid, cerebrospinal fluid (CSF), and peripheral blood during axonal degeneration.^3,4^ Neurofilament proteins are heteropolymers composed of a family of 5 intermediate filaments.^3,4,5^ The largest of these is the neurofilament heavy chain (NfH), followed by the medium chain (NfM), the light chain (NfL), α-internexin, and peripherin.^3,4,5^ A common structure among NfH, NfM, and NfL consists of 3 components: a head domain at the amino-terminal end, a central α-helical rod domain, and a tail domain at the carboxy-terminal end.^3,5^ The NfH and NfM proteins are unique among these 3 NFs in that they have a long carboxy-terminal domain with multiple LysSer-Pro repeats.^3,6^ All 3 NFs can be phosphorylated on their head domain, but only NfH and NfM can be extensively phosphorylated on their carboxy-terminal domain.^3,4,6^ This phosphorylation on the carboxy terminus increases the resistance of NfH and NfM to proteases.^3,4,6^ In addition, the carboxyl terminals of NfH and NfM interact with mitochondria,^7^ and mitochondrial dysfunction in the dopaminergic neurons of the substantia nigra is a hallmark of PD.^8^

Among NFs, NfL has been extensively studied as a prognostic biomarker in PD.^3,4^ Levels of NfL were significantly higher in patients with PD than in heathy controls, and baseline NfL levels were associated with motor and cognitive progression in PD.^9,10,11,12,13,14,15^ Increased NfH levels have also been found in many other neurologic disorders, such as multiple system atrophy, progressive supranuclear palsy, and amyotrophic lateral sclerosis.^16,17,18,19^ Levels of cNfH were significantly higher in the more rapidly progressive syndromes progressive supranuclear palsy and multiple system atrophy than in PD.^16^ Higher cNfH and blood NfH levels at baseline were associated with rapid progression of disease in patients with amyotrophic lateral sclerosis.^17,18,19,20^ However, the association between the levels of NfH and clinical progression in patients with PD remains unclear.

Therefore, this study’s primary aim was to examine whether levels of cNfH are associated with clinical progression of PD in terms of both motor and cognitive symptoms. Two secondary aims were included: to examine whether levels of cNfH correlate with levels of CSF α-synuclein, amyloid-β (1-42) (Aβ_42_), phosphorylated tau at threonine 181 position (P-tau), total tau (T-tau), and magnetic resonance imaging measures; and to compare the associations between NfH, NfL, and clinical progression in the early stages of PD.

## Methods

### Participants

The Parkinson Progression Markers Initiative (PPMI) is a prospective, longitudinal, observational, international multicenter study that aims to identify biomarkers for the progression of PD.^21,22^ Criteria for enrollment were age 30 years or older, within 2 years of diagnosis, Hoehn and Yahr stage less than 3, no treatment with PD medications. The Hoehn and Yahr scale includes stages 1 through 5 (1, unilateral involvement only usually with minimal or no functional disability; 2, bilateral or midline involvement without impairment of balance; 3, bilateral disease [mild to moderate disability with impaired postural reflexes, physically independent]; 4, severely disabling disease; still able to walk or stand unassisted; and 5, confinement to bed or wheelchair unless aided). In addition, at the time of enrollment, patients were required to have either a single asymmetric resting tremor or asymmetric bradykinesia or at least 2 of the following: resting tremor, bradykinesia, or rigidity (must have either resting tremor or bradykinesia).^21,22^ The diagnosis of PD was confirmed with dopamine transporter imaging using single-photon emission computed tomography. This study was approved by the institutional review board at each site, and participants provided written informed consent. This study is reported following the Strengthening the Reporting of Observational Studies in Epidemiology (STROBE) reporting guideline. This cohort study used data from the Parkinson Progression Marker Initiative ranging from June 2010 to November 2018. Data were acquired on April 18, 2021, and analysis was conducted from October 20 to December 18, 2021.

### Clinical Assessments

The Hoehn and Yahr scale is used to describe the symptom progression of Parkinson disease. Motor and nonmotor symptom severity were assessed with the Movement Disorder Society–sponsored revision of the Unified Parkinson Disease Rating Scale (MDS-UPDRS) Parts I, II, and III (scores range from 0 to 132, with higher scores indicating worse motor function).^23^ Cognitive functioning was assessed in several domains, including global cognition, using Montreal Cognitive Assessment (MoCA) (scores range from 0 to 30, with higher scores indicating better cognitive function)^24^; visuospatial function using Benton Judgment of Line Orientation (JLO)^25^; cognitive processing speed using the Symbol Digit Modalities (SDM) test^26^; verbal learning and memory using the Hopkins Verbal Learning Test (HVLT)^27^; executive function using the Semantic Fluency Test (SFT)^28^; and attention and working memory using Letter Number Sequencing (LNS).^29^ Patients with PD received evaluations with the MDS-UPDRS at baseline, every 3 months during the first year, and every 6 months during the subsequent 4 years, and received evaluations of cognitive function at baseline and every year during the subsequent 4 years. Patients with baseline data on cNfH levels and demographic and clinical variables were included. A total of 404 patients with PD were included from the PPMI cohort (Table 1). Patients with missing data during follow-up visits had age, sex, disease duration, and baseline MDS-UPDRS Part III and MoCA scores similar to those of patients without missing data (eTable 1 in the Supplement). The sample sizes of follow-up visits are provided in eTable 2 in the Supplement.

**Table 1.** Baseline Characteristics of the Study Participants

Characteristic	Mean (SD)		*P* value
	Controls (n = 183)	PD (n = 404)	
Age, y	60.6 (11.5)	61.7 (9.7)	.23
Sex, No. (%)			
Female	67 (36.6)	141 (34.9)	.59
Male	116 (63.4)	263 (65.1)	
Education, y	16 (2.9)	15.6 (3.0)	.07
Disease duration, y	NA	0.56 (0.54)	NA
MDS-UPDRS scores			
Part I	3.0 (2.9)	5.5 (4)	<.001
Part II	0.5 (1.0)	5.8 (4.1)	<.001
Part III	1.2 (2.2)	20.9 (8.8)	<.001
Cognitive function			
MoCA score	28.2 (1.1)	27.1 (2.3)	<.001
Benton judgment of line orientation	13.1 (2.0)	12.7 (2.1)	.08
HVLT total recall	26.1 (4.6)	24.5 (5.0)	<.001
Letter number sequencing	10.9 (2.6)	10.6 (2.6)	.13
Semantic fluency test	52.0 (11.1)	48.8 (11.7)	.002
Symbol digit modalities test	46.9 (10.6)	41.2 (9.7)	<.001
CSF biomarkers			
NfH, log_2_ RFU	9.2 (0.7)	9.3 (0.6)	.04
Aβ_42_, pg/mL	1018.3 (505.9)	907.8 (411.3)	.006
α-synuclein, pg/mL	1684.1 (689.3)	1505.4 (670)	.003
P-tau, pg/mL	17.5 (8.5)	14.9 (5.3)	<.001
T-tau, pg/mL	191.7 (80.5)	169.5 (57.1)	<.001

Abbreviations: Aβ_42_, amyloid-β 1-42; CSF, cerebrospinal fluid; HVLT, Hopkins Verbal Learning Test; MDS-UPDRS, Movement Disorder Society–sponsored revision of the Unified Parkinson Disease Rating Scale; MoCA, Montreal Cognitive Assessment; NA, not applicable; NfH, neurofilament heavy; P-tau, phosphorylated tau at threonine 181; RFU, relative fluorescence units; T-tau, total tau.

### CSF and Serum Biomarkers

Cerebrospinal fluid sample collection, handling, shipment, and storage were implemented according to the PPMI biologics manual.^21^ The levels of Aβ_42_, T-tau, and P-tau in CSF were measured using a multiplex platform (xMAP, Luminex Corp) with research use-only immunoassay kit–based reagents (INNO-BIA AlzBio3, Fujirebio-Innogenetics). The concentration of α-synuclein was measured using a commercially available sandwich enzyme-linked immunosorbent assay (BioLegend).

The levels of NfH and NfL in CSF were analyzed using the slow off-rate modified aptamers platform. The relative fluorescence units (RFU) were transformed to a log_2_ scale and normalized to the global median (across all plates) separated by dilution level. The data set was adjusted for batch effects using empirical Bayes methods as implemented in the ComBat function in the R package sva.^30^ Serum NfL protein was measured on the Simoa singleplex NF-light assay (Quanterix). Baseline CSF and serum biomarkers were used in this study.

### Statistical Analysis

The primary goals of this post hoc analysis were the evaluation of the associations between baseline cNfH levels and longitudinal change in motor function (MDS-UPDRS Part III) and cognition (MoCA). Linear mixed-effects models were used to examine the associations between baseline cNfH levels and longitudinal change in clinical scores by examining the interaction of cNfH levels and time since symptom onset. Separate models were fitted for each clinical score, with adjustment for age, sex, race (parent-declared race), educational level, and study site as fixed effects. Participant-specific slope and intercept were modeled as random effects. Missing values in follow-up were not included unless otherwise specified. In additional models, missing data were imputed using the nearest-neighbor method (k = 5) to explore the associations between missing data and the primary outcome. As secondary analyses, sensitivity analyses were performed for MDS-UDPRS Part III and MoCA scores, including individuals who had at least 1, 2, and 3 years of follow-up. In addition, the associations between baseline cNfH levels and longitudinal changes in MDS-UPDRS Part I, MDS-UPDRS Part II scores, and domain-specific cognitive measures (JLO, HVLT, LNS, SFT, and SDM) were evaluated using linear mixed-effects models.

Differences between patients with PD and controls for continuous demographic and clinical variables were assessed using multivariable linear regression models, by converting categorical variables into dummy variables. Differences of categorical demographic characteristics were computed using a χ^2^ test. Associations between cNfH levels and clinical and magnetic resonance imaging measures (eAppendix in the Supplement) were calculated using partial Spearman rank correlation. Age, sex, race, educational level, and study site were adjusted in regression and correlation analysis. Continuous variables are reported as the mean (SD), and categorical variables were summarized using frequencies. All tests were 2-sided with a significance level of *P* < .05. Statistical analyses were performed using MATLAB R2018b (MathWorks), with linear mixed-effects analysis performed using the *fitlme* function.

## Results

### Demographic and Clinical Variables

The demographic and clinical characteristics of the participants included in this study are summarized in Table 1. Although heathy individuals serving as controls were not stringently selected to match patients with PD,^22^ the 2 groups did not differ significantly in age or sex (Table 1), which is similar to previous studies of this cohort at baseline.^22,31^ The mean (SD) age of 404 patients with PD at baseline was 61.7 (9.7) years, 263 were men (65.1%), and 141 were women (34.9%). Parkinson disease had been newly diagnosed and the patients were drug naive, with a median (SD) disease duration of 0.36 (0.56) years (range, 0.03-2.98 years) since symptom onset. The median (SD) follow-up time was 5.26 (1.34) years (range, 0.11-7.99 years). The mean (SD) levels of cNfH were significantly higher in patients with PD compared with the controls (control: 9.2 [0.72] log_2_ RFU vs PD: 9.3 [0.60] log_2_ RFU; *P* = .04) (Table 1).

### Correlation of cNfH Levels, Demographic Characteristics, and Symptoms

Before longitudinal analyses, we examined the cross-sectional associations between cNfH levels, demographic characteristics, and clinical assessments. The cNfH levels correlated with age in both the control and PD groups adjusted for sex (control: *r* = 0.66; 95% CI, 0.57-0.73; *P* < .001; PD: *r* = 0.58; 95% CI, 0.52-0.65; *P* < .001). The mean (SD) levels of cNfH were significantly higher in men vs women with adjustment for age among both the control and PD cohorts (control: 9.10 [0.55] log_2_ RFU vs 8.88 [0.52] log_2_ RFU; *P* = .008; PD: 9.12 [0.45] log_2_ RFU vs 8.91 [0.47] log_2_ RFU; *P* < .001). We next examined whether cNfH levels were correlated with baseline disease severity in patients with PD. The cNfH levels unadjusted for age and sex were significantly correlated with MDS-UPDRS Part III (*r* = 0.21; 95% CI, 0.11-0.30; *P* < .001) and MoCA (*r* = −0.19; 95% CI, −0.28 to −0.10; *P* < .001). These correlations were not found after adjustment for age and sex (MDS-UPDRS Part III: *r* = 0.09; 95% CI, −0.02 to 0.18; *P* = .10; MoCA: *r* = −0.04; 95% CI, −0.14 to 0.05; *P* = .41) (eTable 3 in the Supplement).

### Association of Baseline cNfH Levels With Progression of PD

In linear mixed-effects models controlling for potential confounders, including age, sex, race, educational level, and study site, the MDS-UPDRS Part III score increased by approximately 1.8 units per year (β = 1.83; 95% CI, 1.57-2.10; *P* < .001) and the MoCA score decreased by approximately 0.15 units per year (β = −0.15; 95% CI, −0.22 to −0.08; *P* < .001) (eTable 4 in the Supplement). To examine the associations between cNfH levels and motor and cognitive progression of PD, time-by-cNfH interaction was added in linear mixed-effects models as a fixed effect. The interactions of cNfH levels and time were significant in both models, indicating that higher cNfH levels were associated with a greater increase in MDS-UPDRS Part III scores (β = 0.39; 95% CI, 0.12-0.66; *P* = .003) and a faster decrease in the MoCA score (β = −0.18; 95% CI, −0.24 to −0.11; *P* < .001) (Table 2). These findings were also significant after imputing missing data (Table 2). We also performed sensitivity analyses, including individuals with at least 1-year, 2-year, and 3-year follow-up visits. In these analyses, cNfH levels were also significantly associated with the annual rate of change in the MDS-UPDRS Part III and MoCA scores, and the findings were similar to those in the primary group (Table 2). In subanalyses, we found baseline cNfH levels were also associated with longitudinal changes in MDS-UPDRS Part I, MDS-UPDRS Part II, and domain-specific cognitive measures, including JLO, HVLT, LNS, SFT, and SDM (eTable 5 in the Supplement).

**Table 2.** Association Between cNfH Levels and Annual Rate of Change in Motor and Cognitive Scores

Models^a^	No.	MDS-UPDRS Part III score		MoCA score	
		β (95% CI)	*P* value	β (95% CI)	*P* value
Primary model	404	0.39 (0.12 to 0.66)	.003	−0.18 (−0.24 to −0.11)	<.001
Model 1	404	0.28 (0.05 to 0.52)	.02	−0.18 (−0.24 to −0.11)	<.001
Model 2	388	0.38 (0.13 to 0.65)	.004	−0.17 (−0.24 to −0.11)	<.001
Model 3	376	0.38 (0.12 to 0.64)	.005	−0.17 (−0.24 to −0.11)	<.001
Model 4	366	0.36(−0.10 to 0.63)	.007	−0.14 (−0.22 to −0.08)	<.001

Abbreviations: cNfH, cerebrospinal neurofilament heavy; MDS-UPDRS, Movement Disorder Society–sponsored revision of the Unified Parkinson Disease Rating Scale; MoCA, Montreal Cognitive Assessment.

^a^ Each row shows the results for 2 terms from a single mixed-effects model with clinical score as the outcome and showing the interaction between cNfH levels and time. All linear mixed-effects models included age, sex, race, educational level, study site, cNfH levels, time since symptom onset, and the interaction of cNfH levels with time as fixed effects. Missing values in follow-up visits were not included in the primary model. Missing values in follow-up visits were imputed using nearest-neighbor method in model 1. Model 2 included individuals with at least 1 year of follow-up visits, model 3 included individuals with at least 2 years of follow-up visits, and model 4 included individuals with at least 3 years of follow-up visits.

**Figure 1.**
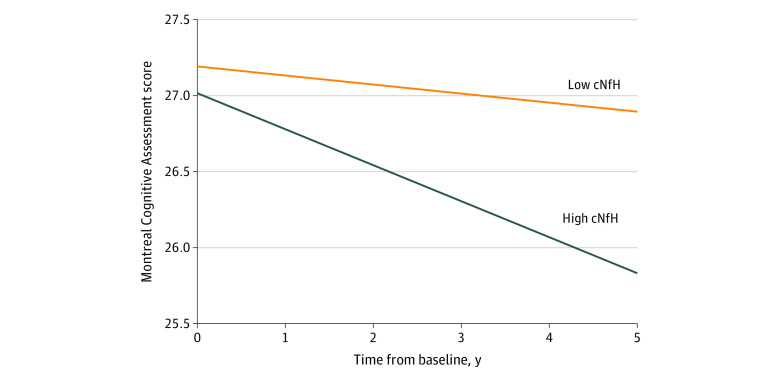
Longitudinal Trajectories of Mean Montreal Cognitive Assessment in Patients With Parkinson Disease With High or Low Cerebrospinal Fluid Neurofilament Heavy (cNfH) Levels Montreal Cognitive Assessment scores range from 0 to 30, with higher scores indicating better cognitive function.

To further support the association between baseline cNfH levels and clinical progression of PD, clinical trajectories were examined in patients with low and high levels of baseline cNfH. The groups with low and high levels of cNfH were created using the mean cNfH level (8.97 log_2_ RFU, adjusted for covariates) in the group as the cutoff value. Linear mixed-effect models were implemented to compare the progression of PD in the 2 groups (n = 214 for low cNfH level group; n = 190 for high cNfH level group) by testing the interaction between group and time. There was a significant difference in the rate of cognitive decline, measured by the MoCA (group × time: β = −0.16; 95% CI, −0.30 to −0.02; *P* = .02) (Figure 1). The rate of change of the MDS-UPDRS Part III score (group × time: β = 0.40; 95% CI, −0.13 to 0.92; *P* = .14) did not significantly differ between the low and high cNfH level groups (eTable 6 in the Supplement).

### Association of cNfH Levels With Other CSF Biomarkers

**Figure 2.**
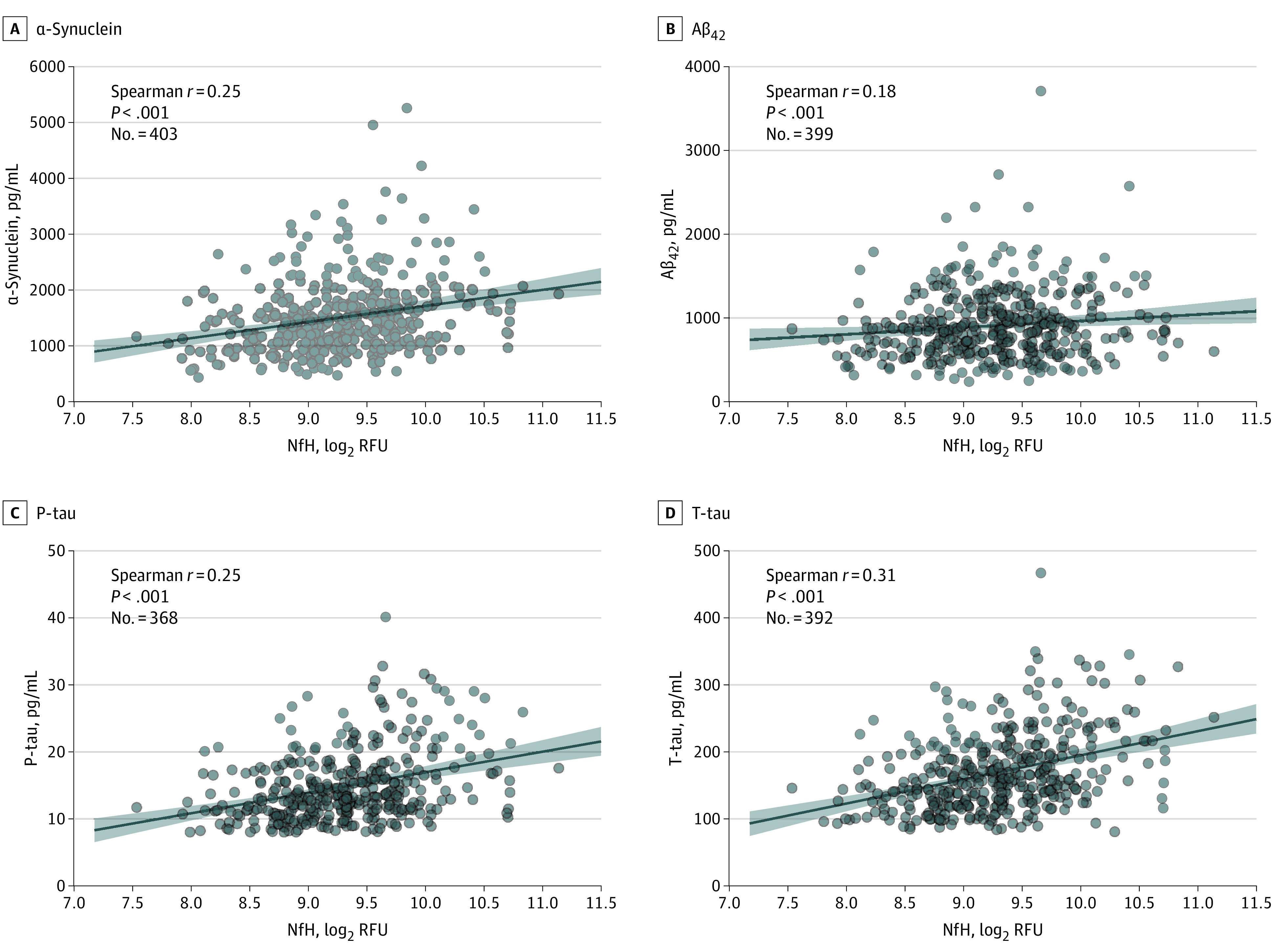
Correlations of Cerebrospinal Fluid (CSF) Neurofilament Heavy (cNfH) Levels With CSF Biomarkers at Baseline Levels of baseline cNfH and the CSF biomarkers α-synuclein (A), amyloid-β (1-42) (Aβ_42_) (B), phosphorylated tau at threonine 181 position (P-tau) (C), and total tau (T-tau) (D) were adjusted for all covariates. Shaded regions represent 95% CIs for the fit of each linear regression model. RFU indicates relative fluorescence units.

We examined the cross-sectional association between baseline levels of cNfH and 4 other CSF biomarkers (ie, α-synuclein, Aβ_42_, P-tau, and T-tau) among patients with PD. We found positive correlations between the levels of cNfH and these biomarkers with adjustment for all covariates (cNfH vs α-synuclein [n = 403]: *r* = 0.25; 95% CI, 0.15-0.34; *P* < .001; cNfH vs Aβ_42_ [n = 399]: *r* = 0.18; 95% CI, 0.08-0.27; *P* < .001; cNfH vs P-tau [n = 368]: *r* = 0.25; 95% CI, 0.15-0.35; *P* < .001; cNfH vs T-tau [n = 392]: *r* = 0.31; 95% CI, 0.21-0.40; *P* < .001) (Figure 2).

### Association of cNfH Levels With Clinical Progression vs CSF and Serum NfL

Levels of cNfH were significantly associated with both CSF and serum NfL levels after correction for age and sex (CSF NfL: *r* = 0.58; 95% CI, 0.50-0.65; *P* < .001; serum NfL: *r* = 0.29; 95% CI, 0.18-0.40; *P* < .001) (eFigure in the Supplement). To avoid collinearity issues, we fitted separate linear mixed-effects models for each biomarker with each clinical score. A subset of patients with PD (n = 358) with baseline cNfH, CSF NfL, and serum NfL data were included in these analyses. In these subpopulation analyses, cNfH levels were associated with longitudinal changes in the MDS-UPDRS Part III (β = 0.45; 95% CI, 0.17-0.73; *P* = .002) and MoCA (β = −0.18; 95% CI, −0.25 to −0.11; *P* < .001) scores (eTable 7 in the Supplement). Cerebrospinal fluid NfL levels were not associated with longitudinal changes in the MDS-UPDRS Part III (β = 0.10; 95% CI, −0.18-0.39; *P* = .48) and MoCA (β = −0.05; 95% CI, −0.13 to −0.02; *P* = .14) scores. Serum NfL levels were not associated with longitudinal changes in MDS-UPDRS Part III scores (β = 0.20; 95% CI, −0.08 to 0.48; *P* = .17). Serum NfL levels were associated with longitudinal changes in MoCA (β = −0.15; 95% CI, −0.23 to −0.08; *P* < .001), but the standardized β coefficients were lower compared with the cNfH levels (serum NfL: standardized β = −4.15; cNfH: standardized β = −4.91). These results suggest that baseline cNfH level may be more informative for clinical progression compared with NfL level in the CSF or serum in the early stages of PD.

## Discussion

In this study, baseline cNfH levels were associated with longitudinal changes in motor and cognitive scores and baseline CSF biomarker levels in patients with PD. In addition, we found that the cNfH level was more informative for the longitudinal change in motor and cognitive scores compared with the NfL level. These results support the potential utility of cNfH levels in clinical trials by enrichment of patients with PD who have faster disease progression rates.

Our findings extend existing knowledge by showing that cNfH levels could reflect both cognitive and motor aspects of PD progression. This finding is in line with previous observations that NfH levels were associated with clinical progression in amyotrophic lateral sclerosis.^17,18,19,20^ Cognitive impairment in PD is heterogeneous, and single or multiple cognitive domains can be affected in patients with PD who have cognitive impairment.^32^ In the present study, cNfH levels were associated not only with decline in global cognition (MoCA), but also decline in domain-specific cognition, including visuospatial function (JLO), cognitive processing speed (SDM), verbal learning and memory (HVLT), executive function (SFT), and attention and working memory (LNS). In addition, we found higher cNfH levels were associated with faster progression of nonmotor (MDS-UPDRS Part I) and motor (MDS-UPDRS Part II) experiences of daily living. Thus, the association between cNfH levels and clinical progression of PD was not domain-specific.

Baseline cNfH levels positively correlated with levels of α-synuclein, Aβ_42_, P-tau, and T-tau in CSF, suggesting that pathologic changes in these biomarkers may lead to axonal degeneration, releasing neurofilaments into the CSF, or coexistence of these pathologic findings in PD.^33^ These results are consistent with reports that levels of CSF proteins correlated with each other^31,34^ and that higher levels of α-synuclein and P-tau were associated with faster progression of PD.^31,35^ In addition, we found a negative association between cNfH levels and the volume of left choroid plexus (eAppendix and eTable 8 in the Supplement), which aligns with a previous study.^36^ The prognostic value of cNfH levels is also supported by the observed correlations between levels of cNfH and NfL in the CSF and serum (eFigure in the Supplement). Future work combining multiple fluid biomarkers may help improve the prognostic estimation of PD progression.^33^

A possible interpretation of the difference in the β coefficients is that posttranslational modifications affect the degradation of neurofilaments.^4^ For example, phosphorylation on carboxyl terminal domains of NfH and NfM increases their resistance to protease cleavage.^3,6^ Considering that NfM colocalizes with the dopamine D_1_ receptor in synapses,^6^ the prognostic value of the NfM level in PD should be explored.

### Limitations

This study has limitations. Our results must be interpreted in the context of the study population and the use of CSF rather than blood. The PPMI enrolled patients with early-stage PD who were younger and had less baseline disability than the general population of patients with PD. Therefore, the PPMI data set is not representative of the natural history of PD progression.^21,22,37^ In addition, NfL may be more informative for PD progression in older patients, and further work is needed to understand which NFs are most informative for patients with PD of different ages. The levels of cNfH levels were higher in patients with PD compared with controls, which is consistent with previous findings of elevated cNfH levels in patients with atypical parkinsonism compared with controls.^16^ However, the difference is not very large in this study, which may limit the usefulness of cNfH levels in the diagnosis of PD. Another limitation is that this was a single cohort study; therefore, our findings should be validated in other cohorts, even though the PPMI study enrolled participants from multiple sites.

## Conclusions

In this post hoc cohort study, cNfH levels were associated with clinical progression and baseline levels of CSF biomarkers (ie, α-synuclein, Aβ_42_, P-tau, and T-tau) in patients with PD. Our findings suggest that cNfH level measurements might assist clinicians in identifying patients with PD at risk of fast clinical progression. Further study is warranted to assess the associations of NfH levels in blood and clinical progression in PD.

## Notes

Supplement. **eAppendix.** MRI Acquisition and Preprocessing, and Results of Correlation Analysis **eTable 1.** Comparisons of Baseline Demographics and Clinical Variables of Patients With and Without Missing Data During Follow-up Visits **eTable 2.** The Sample Size of Follow-up Visits **eTable 3.** Correlations Between cNfH Levels and Baseline Clinical Measures **eTable 4.** Longitudinal Evolution of Clinical Variables in Patients With Parkinson Disease **eTable 5.** Association Between Baseline cNfH Levels and Annual Rate of Change in Clinical Measures **eTable 6.** Linear Mixed Effects Models Examining Disease Progression in Patients With High and Low cNfH Levels **eTable 7.** Association Between Neurofilaments Levels and Annual Rate of Change in Clinical Measures **eTable 8.** Correlations Between cNfH Levels and the Volumes of Brain Regions **eFigure.** Correlations Between cNfH Levels and Levels of NfL in CSF and Serum
