# Supplementary material for: Association of Cerebrospinal Fluid Neurofilament Heavy Protein Levels With Clinical Progression in Patients With Parkinson Disease
Source: JAMA Netw Open. 2022 Jul 26;5(7):e2223821. doi: 10.1001/jamanetworkopen.2022.23821 (PMC9327574; doi:10.1001/jamanetworkopen.2022.23821)
Supplement: Supplement. — eAppendix. MRI Acquisition and Preprocessing, and Results of Correlation Analysis eTable 1. Comparisons of Baseline Demographics and Clinical Variables of Patients With and Without Missing Data During Follow-up Visits eTable 2. The Sample Size of Follow-up Visits eTable 3. Correlations Between cNfH Levels and Baseline Clinical Measures eTable 4. Longitudinal Evolution of Clinical Variables in Patients With Parkinson Disease eTable 5. Association Between Baseline cNfH Levels and Annual Rate of Change in Clinical Measures eTable 6. Linear Mixed Effects Models Examining Disease Progression in Patients With High and Low cNfH Levels eTable 7. Association Between Neurofilaments Levels and Annual Rate of Change in Clinical Measures eTable 8. Correlations Between cNfH Levels and the Volumes of Brain Regions eFigure. Correlations Between cNfH Levels and Levels of NfL in CSF and Serum [file jamanetwopen-e2223821-s001.pdf]

Supplementary Online Content

Wang L, Zhang W, Liu F, et al. Association of cerebrospinal fluid neurofilament heavy protein levels with clinical progression in patients with Parkinson disease. *JAMA Netw Open*. 2022;5(7):e2223821. doi:10.1001/jamanetworkopen.2022.23821

eAppendix. MRI Acquisition and Preprocessing, and Results of Correlation Analysis

eTable 1. Comparisons of Baseline Demographics and Clinical Variables of Patients With and Without Missing Data During Follow-up Visits

eTable 2. The Sample Size of Follow-up Visits

eTable 3. Correlations Between cNfH Levels and Baseline Clinical Measures

eTable 4. Longitudinal Evolution of Clinical Variables in Patients With Parkinson Disease

eTable 5. Association Between Baseline cNfH Levels and Annual Rate of Change in Clinical Measures

eTable 6. Linear Mixed Effects Models Examining Disease Progression in Patients With High and Low cNfH Levels

eTable 7. Association Between Neurofilaments Levels and Annual Rate of Change in Clinical Measures

eTable 8. Correlations Between cNfH Levels and the Volumes of Brain Regions

eFigure. Correlations Between cNfH Levels and Levels of NfL in CSF and Serum

This supplementary material has been provided by the authors to give readers additional information about their work.

© 2022 Wang L et al. *JAMA Network Open.*

eAppendix. MRI acquisition and preprocessing, and results of correlation analysis

MRI acquisition and preprocessing

T1-weighted MRI scans were acquired according to the PPMI MRI Technical Operations Manual(https://www.ppmi-info.org/)[1]. Acquisition parameters were as follows: repetition time = 2300/1900 ms; echo time = 2.98/2.96/2.27/2.48/2.52 ms; inversion time = 900 ms; flip angle =9°; matrix = 240 × 256/256 × 256; and voxel = 1 × 1 × 1 mm^3.

Brain reconstruction and volumetric segmentation was performed with the FreeSurfer version 6.0, which is documented and freely available for download online (http://surfer.nmr.mgh.harvard.edu/). The technical details of these procedures are described in prior publications[2–10]. In brief, this processing includes motion correction, removal of non-brain tissue using a hybrid watershed/surface deformation procedure, automated Talairach transformation, segmentation of the subcortical white matter and deep gray matter volumetric structures intensity normalization, tessellation of the gray matter white matter boundary, automated topology correction, and surface deformation following intensity gradients to optimally place the gray/white and gray/cerebrospinal fluid borders at the location where the greatest shift in intensity defines the transition to the other tissue class. Regional volumes were quantified according the 2010 Desikan-Killany atlas. Acquisition site and total intracranial volume were regressed out before correlation analyses. We focused on subcortical regions (i.e., accumbens, amygdala, caudate, hippocampus, pallidum, putamen, thalamus) and choroid plexus (ChP). Previous analyses have shown that subcortical atrophy occur early in Parkinson's disease[11,12], while the ChP is the dominant source of CSF production and may contribute to the clearance of CSF proteins[13–15].

cNfH levels are correlated with the volumes of left choroid plexus and right putamen

To test whether cNfH levels are related to brain atrophy, we correlated the levels of cNfH with imaging measures. Among above mentioned regions, left ChP (r = -0.17, CI -0.31 to -0.11, *P* = 0.02 FDR corrected) and right putamen (r = -0.17, CI -0.27 to -0.05, *P* = 0.02 FDR corrected) survived corrections for multiple comparisons (eTable 8). The negative association between cNfH level and left ChP volume aligns with a previous study finding that ChP volume is negatively associated with levels of CSF proteins[13].

© 2022 Wang L et al. *JAMA Network Open.*

eTable 1. Comparisons of baseline demographics and clinical variables of patients with and without missing data during follow up visits.

Variables	Without missing data (n = 123)	With missing data (n = 281)	*P*-value
Age, yr	61.28 (9.26)	61.93(9.88)	0.54
Male sex (%)	0.68(84)	0.64(179)	0.37
Education, yr	15.5(3.01)	15.61(2.94)	0.73
Disease duration, yr	0.58(0.56)	0.55(0.54)	0.6
**MDS-UPDRS scores**			
MDS-UPDRS Part-I	4.77(3.44)	5.84(4.22)	0.01
MDS-UPDRS Part-II	5.31(4.14)	6.04(4.03)	0.10
MDS-UPDRS Part-III	20.5(8.58)	21.01(8.9)	0.60
**Cognitive function**			
MoCA score	26.98(2.19)	27.17(2.37)	0.44
Benton Judgment of Line Orientation	13.13(2.06)	12.58(2.17)	0.02
HVLT Total Recall	25.25(4.43)	24.15(5.23)	0.04
Letter Number Sequencing	11.02(2.39)	10.41(2.72)	0.03
Semantic Fluency test	49.43(10.59)	48.46(12.13)	0.44
Symbol Digit Modalities test	41.23(10.08)	41.17(9.56)	0.96

Abbreviations: HVLT, Hopkins Verbal Learning Test Discrimination Recognition score; MDS-UPDRS, Movement Disorder Society–sponsored revision of the Unified Parkinson’s Disease Rating Scale; MoCA, Montreal Cognitive Assessment; NfH, neurofilament heavy.

© 2022 Wang L et al. *JAMA Network Open.*

eTable 2. The sample size of follow up visits. Duration is presented as mean (standard deviation).

Follow-up visits	Follow up time (years)	Duration (years)	PD patients
**BL**	0	0.56(0.54)	404
**V02**	0.25	0.80(0.53)	371
**V03**	0.5	1.07(0.54)	269
**V04**	0.75	1.32(0.54)	319
**V05**	1.0	1.56(0.54)	344
**V06**	1.5	2.05(0.54)	353
**V07**	2.0	2.56(0.55)	348
**V08**	2.5	3.06(0.54)	348
**V09**	3.0	3.56(0.56)	348
**V10**	3.5	4.07(0.56)	337
**V11**	4.0	4.56(0.55)	325
**V12**	4.5	5.07(0.57)	301
**V13**	5.0	5.56(0.56)	298

© 2022 Wang L et al. *JAMA Network Open.*

eTable 3. Correlations between cNfH levels and baseline clinical measures. The results for cNfH levels prior to and after adjusting for age and sex are shown. The raw *P*-value are presented.

**Clinical Variables**	**cNfH levels unadjusted for age and sex**		**cNfH levels adjusted for age and sex**	
	Spearman r	*P*-value	Spearman r	*P*-value
**MDS-UPDRS scores**				
MDS-UPDRS Part-I	0.09	0.07	0.11	0.04
MDS-UPDRS Part-II	0.15	**0.003**	0.12	**0.02**
MDS-UPDRS Part-III	0.21	**<0.001**	0.09	0.10
**Cognitive function**				
MoCA score	-0.19	**<0.001**	-0.04	0.41
Benton Judgment of Line Orientation	-0.10	0.06	-0.03	0.53
HVLT Total Recall	-0.26	**<0.001**	0.01	0.83
Letter Number Sequencing	-0.24	**<0.001**	0.01	0.91
Semantic Fluency test	-0.24	**<0.001**	0.01	0.91
Symbol Digit Modalities test	-0.32	**<0.001**	-0.03	0.53

Abbreviations: HVLT, Hopkins Verbal Learning Test Discrimination Recognition score; MDS-UPDRS, Movement Disorder Society–sponsored revision of the Unified Parkinson's Disease Rating Scale; MoCA, Montreal Cognitive Assessment; NfH, neurofilament heavy.

© 2022 Wang L et al. *JAMA Network Open.*

eTable 4. Longitudinal evolution of clinical variables in patients with Parkinson's disease. Longitudinal evolution of clinical variables are described by the estimate and 95% CI of the time fixed effect of the linear mixed-effects models.

**Clinical Variables**	**β (95% CI)**	***P*-value**
**MDS-UPDRS scores**		
MDS-UPDRS Part-I	0.94 (0.83 to 1.05)	**<0.001**
MDS-UPDRS Part-II	0.95 (0.82 to 1.07)	**<0.001**
MDS-UPDRS Part-III	1.83 (1.57 to 2.10)	**<0.001**
**Cognitive function**		
MoCA score	-0.15 (-0.22 to -0.08)	**<0.001**
Benton Judgment of Line Orientation	-0.06 (-0.10 to -0.02)	**0.003**
HVLT Total Recall	-0.07 (-0.16 to 0.03)	0.17
Letter Number Sequencing	-0.15 (-0.20 to -0.10)	**<0.001**
Semantic Fluency test	-0.31 (-0.50 to 0.11)	**0.002**
Symbol Digit Modalities test	-0.60 (-0.79 to -0.40)	**<0.001**

Abbreviations: HVLT, Hopkins Verbal Learning Test Discrimination Recognition score; MDS-UPDRS, Movement Disorder Society–sponsored revision of the Unified Parkinson's Disease Rating Scale; MoCA, Montreal Cognitive Assessment.

© 2022 Wang L et al. *JAMA Network Open.*

e**Table 5. Association between baseline cNfH levels and annual rate of change in clinical measures.**

Clinical Variables	β (95% CI)	*P*-value
**MDS-UPDRS scores**		
MDS-UPDRS Part-I	0.16 (0.06 to 0.27)	**0.003**
MDS-UPDRS Part-II	0.24 (0.12 to 0.37)	**< 0.001**
**Cognitive function**		
Benton Judgment of Line Orientation	-0.06 (-0.10 to -0.02)	**0.005**
HVLT Total Recall	-0.21 (-0.31 to -0.10)	**< 0.001**
Letter Number Sequencing	-0.11 (-0.16 to -0.06)	**<0.001**
Semantic Fluency test	-0.40 (-0.60 to -0.20)	**< 0.001**
Symbol Digit Modalities test	-0.41 (-0.60 to -0.21)	**< 0.001**

Abbreviations: HVLT, Hopkins Verbal Learning Test Discrimination Recognition score; MDS-UPDRS, Movement Disorder Society–sponsored revision of the Unified Parkinson's Disease Rating Scale.

© 2022 Wang L et al. *JAMA Network Open.*

eTable 6. Linear mixed effects models examining disease progression in patients with high and low cNfH levels.

Clinical Variables	β (95% CI)	*P*-value
**MDS-UPDRS scores**		
MDS-UPDRS Part-I	0.003 (-0.21 to 0.21)	0.98
MDS-UPDRS Part-II	0.27 (0.02 to 0.52)	**0.03**
MDS-UPDRS Part-III	0.40 (-0.13 to 0.92)	0.14
**Cognitive function**		
MoCA score	-0.16 (-0.30 to -0.02)	**0.02**
Benton Judgment of Line Orientation	-0.04 (-0.12 to 0.05)	0.41
HVLT Total Recall	-0.17 (-0.38 to 0.04)	0.10
Letter–Number Sequencing	-0.17 (-0.27 to -0.07)	**<0.001**
Semantic Fluency test	-0.53 (-0.92 to -0.13)	**0.009**
Symbol Digit Modalities test	-0.14 (-0.54 to 0.26)	0.49

Abbreviations: HVLT, Hopkins Verbal Learning Test Discrimination Recognition score; MDS-UPDRS, Movement Disorder Society–sponsored revision of the Unified Parkinson's Disease Rating Scale; MoCA, Montreal Cognitive Assessment.

© 2022 Wang L et al. *JAMA Network Open.*

eTable 7. Association between neurofilaments levels and annual rate of change in clinical measures. CSF NfH, CSF NfL and Serum NfL biomarkers were scaled to zero mean and unit variance so that effect sizes are directly comparable (n = 343). CSF NfL were not significantly associated with longitudinal changes in all scores. Serum NfL were also significantly associated with longitudinal changes in MDS-UPDRS Part-I (serum NfL: β =0.13, CI 0.02 to 0.24, *P* = 0.03; cNfH: β = 0.16, CI 0.04 to 0.27, *P* = 0.007;), LNS (serum NfL: β = -0.08, CI -0.13 to -0.02, *P*= 0.004; cNfH: β = -0.10, -0.15 to -0.05, *P* < 0.001) and SFT(serum NfL: β = -0.34,CI -0.55 to -0.13, P = 0.002; cNfH: β = -0.39, CI -0.60 to -0.18, *P* < 0.001), but these associations were stronger for cNfH levels as compared to Serum NfL levels. Serum NfL has stronger association with longitudinal changes in HVLT compared with cNfH (serum NfL: β = -0.17, CI -0.28 to -0.06, standardized β = -3.12, *P* = 0.002; cNfH: β = -0.17, CI -0.28 to -0.06, standardized β = -3.11, *P* = 0.002).

**Clinical Variables**	CSF NfH			CSF NfL			Serum NfL		
	**β (95% CI)**	**Standardize β**	***P***	**β (95% CI)**	**Standardize β**	***P***	**β (95% CI)**	**Standardize β**	***P***
**MDS-UPDRS scores**									
MDS-UPDRS Part-I	0.16 (0.04 to 0.27)	2.72	0.007	0.03 (-0.08 to 0.15)	0.59	0.55	0.13 (0.02 to 0.24)	2.22	0.03
MDS-UPDRS Part-II	0.24 (0.11 to 0.38)	3.6	<0.001	0.07 (-0.06 to 0.21)	1.09	0.28	0.13 (0 to 0.27)	1.98	0.05
MDS-UPDRS Part-III	0.45 (0.17 to 0.73)	3.15	0.002	0.10 (-0.18 to 0.39)	0.71	0.48	0.20 (-0.08 to 0.48)	1.38	0.17
**Cognitive scores**									
MoCA score	-0.18 (-0.25 to -0.11)	-4.91	<0.001	-0.05 (-0.13 to 0.02)	-1.46	0.14	-0.15 (-0.23 to -0.08)	-4.15	<0.001
Benton Judgment of Line Orientation	-0.06 (-0.10 to -0.01)	-2.48	0.01	-0.01 (-0.05 to 0.04)	-0.31	0.75	-0.05 (-0.09 to 0)	-1.99	0.05
HVLT Total Recall	-0.17 (-0.28 to -0.06)	-3.11	0.002	-0.07 (-0.18 to 0.04)	-1.28	0.20	-0.17 (-0.28 to -0.06)	-3.12	0.002
Letter–Number Sequencing	-0.10 (-0.15 to -0.05)	-3.76	<0.001	-0.03 (-0.08 to 0.03)	-0.94	0.35	-0.08 (-0.13 to -0.02)	-2.87	0.004
Semantic Fluency test	-0.39 (-0.60 to -0.18)	-3.69	<0.001	-0.14 (-0.35 to 0.07)	-1.27	0.21	-0.34 (-0.55 to -0.13)	-3.19	0.002
Symbol Digit Modalities test	-0.4 (-0.60 to -0.21)	-4.01	<0.001	-0.03 (-0.23 to 0.17)	-0.26	0.79	-0.21 (-0.41 to -0.01)	-2.08	0.04

Abbreviations: HVLT, Hopkins Verbal Learning Test Discrimination Recognition score; MDS-UPDRS, Movement Disorder Society–sponsored revision of the Unified Parkinson's Disease Rating Scale; MoCA, Montreal Cognitive Assessment.

© 2022 Wang L et al. *JAMA Network Open.*

eTable 8. Correlations between cNfH levels and the volumes of brain regions. Covariates including age, sex, race, years of education and site effects were adjusted. The q-value are corrected for multiple comparisons using the Benjamini-Hochberg procedure.

Brian regions	r-values	*P*-value	*q*-value
Left_Thalamus	0.05 (-0.06 to 0.16)	0.39	0.66
Left_Caudate	-0.06 (-0.18 to 0.05)	0.25	0.66
Left_Putamen	-0.13 (-0.25 to -0.03)	0.02	0.10
Left_Pallidum	0.02 (-0.07 to 0.13)	0.65	0.75
Left_Amygdala	-0.02 (-0.15 to 0.06)	0.66	0.75
Left_Hippocampus	-0.10 (-0.17 to 0.02)	0.06	0.18
Left_Accumbens	-0.03 (-0.08 to 0.16)	0.65	0.75
Left_choroid_plexus	-0.17 (-0.31 to -0.11)	0.002	**0.02**
Right_Thalamus	0.003 (-0.10 to 0.13)	0.95	0.95
Right_Caudate	-0.05 (-0.17 to 0.08)	0.41	0.66
Right_Putamen	-0.17 (-0.28 to -0.05)	0.002	**0.02**
Right_Pallidum	0.05 (-0.04 to 0.16)	0.39	0.66
Right_Amygdala	-0.04 (-0.17 to 0.05)	0.46	0.66
Right_Hippocampus	-0.06 (-0.13 to 0.09)	0.32	0.66
Right_Accumbens	-0.01 (-0.06 to 0.18)	0.81	0.86
Right_choroid_plexus	-0.11 (-0.27 to -0.06)	0.04	0.17

© 2022 Wang L et al. *JAMA Network Open.*

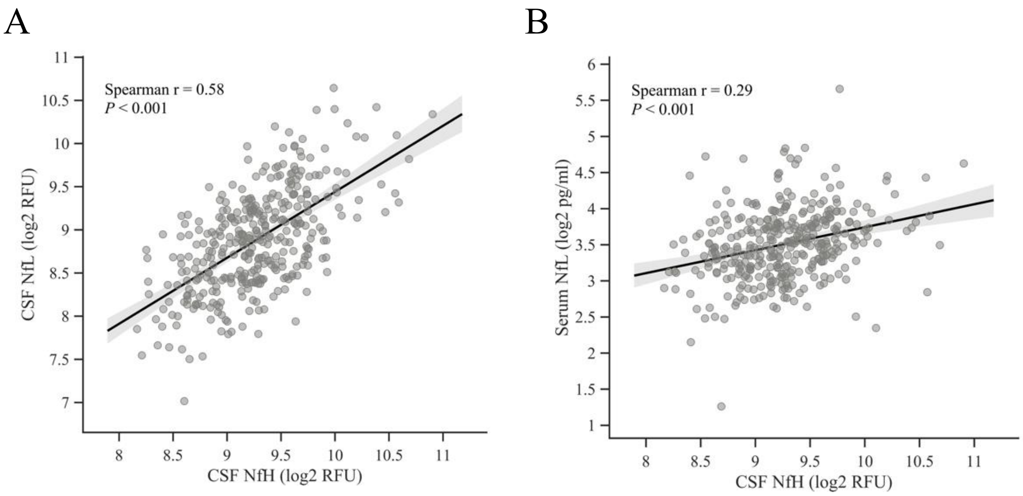

eFigure 1. Correlations between cNfH levels and levels of NfL in CSF and serum. Covariates including age, sex, race, years of education and site effects were adjusted.

© 2022 Wang L et al. *JAMA Network Open.*

eReferences

1. Marek K, Jennings D, Lasch S, et al. The Parkinson Progression Marker Initiative (PPMI). *Prog Neurobiol*. 2011;95(4):629-635. doi:10.1016/j.pneurobio.2011.09.005

2. Fischl B, Salat DH, van der Kouwe AJW, et al. Sequence-independent segmentation of magnetic resonance images. *Neuroimage*. 2004;23(Supplement 1):S69-S84. doi:DOI: 10.1016/j.neuroimage.2004.07.016

3. Fischl B, Salat D ~H., Busa E, et al. Whole brain segmentation: automated labeling of neuroanatomical structures in the human brain. *Neuron*. 2002;33:341-355.

4. Dale A, Fischl B, Sereno MI. Cortical Surface-Based Analysis: I. Segmentation and Surface Reconstruction. *Neuroimage*. 1999;9(2):179-194. doi:10.1006/nimg.1998.0395

5. Fischl B, Dale AM. Measuring the thickness of the human cerebral cortex from magnetic resonance images. *Proc Natl Acad Sci U S A*. 2000;97(20):11050-11055.

6. Fischl B, Liu A, Dale A ~M. Automated manifold surgery: constructing geometrically accurate and topologically correct models of the human cerebral cortex. *{IEEE} Med Imaging*. 2001;20(1):70-80.

7. Reuter M, Schmansky NJ, Rosas HD, Fischl B. Within-Subject Template Estimation for Unbiased Longitudinal Image Analysis. *Neuroimage*. 2012;61(4):1402-1418. doi:10.1016/j.neuroimage.2012.02.084

8. Reuter M, Rosas HD, Fischl B. Highly Accurate Inverse Consistent Registration: A Robust Approach. *Neuroimage*. 2010;53(4):1181-1196. doi:10.1016/j.neuroimage.2010.07.020

9. Fischl B, van der Kouwe A, Destrieux C, et al. Automatically Parcellating the Human Cerebral Cortex. *Cereb Cortex*. 2004;14(1):11-22. doi:10.1093/cercor/bhg087

10. Reuter M, Rosas HD, Fischl B. Accurate Inverse Consistent Robust Registration. *Neuroimage*. 2010;53(4):1181-1196.

11. Zeighami Y, Ulla M, Iturria-Medina Y, et al. Network structure of brain atrophy in de novo parkinson's disease. *Elife*. 2015;4(September 2015):1-20. doi:10.7554/eLife.08440

12. Pandya S, Zeighami Y, Freeze B, et al. Predictive model of spread of Parkinson's pathology using network diffusion. *Neuroimage*. 2019;192(March):178-194. doi:10.1016/j.neuroimage.2019.03.001

13. Tadayon E, Pascual-Leone A, Press D, Santarnecchi E. Choroid plexus volume is associated with levels of CSF proteins: relevance for Alzheimer's and Parkinson's disease. *Neurobiol Aging*. 2020;89:108-117. doi:10.1016/j.neurobiolaging.2020.01.005

14. Xu H, Fame RM, Sadegh C, et al. Choroid plexus NKCC1 mediates cerebrospinal fluid clearance during mouse early postnatal development. *Nat Commun*. 2021;12(1):1-16. doi:10.1038/s41467-020-20666-3

© 2022 Wang L et al. *JAMA Network Open.*

15. Eide PK, Valnes LM, Pripp AH, Mardal KA, Ringstad G. Delayed clearance of cerebrospinal fluid tracer from choroid plexus in idiopathic normal pressure hydrocephalus. *J Cereb Blood Flow Metab*. 2020;40(9):1849-1858. doi:10.1177/0271678X19874790

© 2022 Wang L et al. *JAMA Network Open.*
